# Draft Genome of *Kangiella* sp. Strain TOML190, Isolated from the Surface of the Striped Shore Crab, Pachygrapsus crassipes

**DOI:** 10.1128/mra.00437-22

**Published:** 2022-08-04

**Authors:** Abdullah Adham Zulmajdi, Tatsuru Okazaki, Tetsushi Mori

**Affiliations:** a Department of Biotechnology and Life Science, Tokyo University of Agriculture and Technology, Koganei, Tokyo, Japan; Montana State University

## Abstract

*Kangiella* sp. strain TOML190 is a strain from the *Kangiella* genus that was isolated from the surface of a crustacean. Genetic background analysis of this strain shows that it harbors unique features possibly related to its symbiotic adaptation to its residing host.

## ANNOUNCEMENT

**FIG 1:**
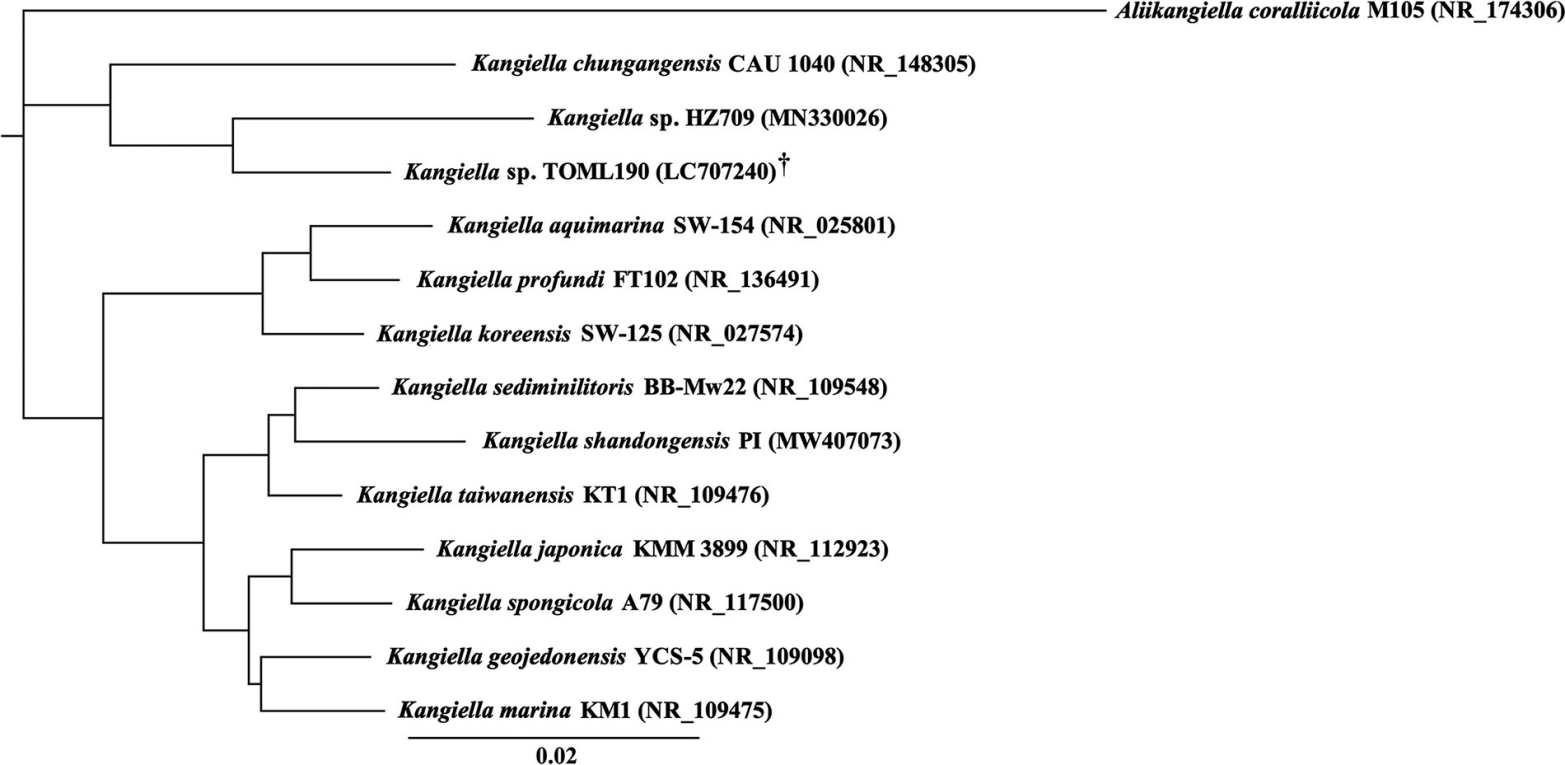
16S rRNA gene similarity of *Kangiella* sp. TOML190 (†) with currently reported *Kangiella* strains. The 16S rRNA gene of TOML190 was extracted from the draft genome presented in this work. The phylogenetic tree was generated using Geneious Prime v2020.2.5 based on the neighbor-joining tree and Tamura-Nei genetic distance model. The scale bar represents a 2% nucleotide sequence difference. *Kangiella* sp. HZ709 has not been published but was included in the tree generation due to its highest similarity to strain TOML190. Aliikangiella coralliicola was used as the outgroup. Accession numbers are derived from recently curated versions of the gene sequences.

*Kangiella* are unique for their ability to produce iso-branched fatty acids (1–3) and have been used to understand bacterial genome reduction (4). Thus far, 11 strains have been reported (3), and a majority of the strains were isolated from marine environments, apart from Kangiella spongicola (5). Here, we present the draft genome sequence of *Kangiella* sp. strain TOML190, a strain showing 96.9% 16S rRNA gene similarity to its closest relative, *Kangiella* sp. strain HZ709 (GenBank number MN330026.1) (Fig. 1). This bacterium is unique in this genus, as it was isolated from the surface of the striped shore crab, Pachygrapsus crassipes.

Upon collection from Ohama Beach, Kanagawa, Japan, in 2020, *P. crassipes* was immediately rinsed with filter-sterilized seawater to remove surface contaminants. A dental water flosser was used to vigorously detach microbes from the crab’s surface, and the collected supernatant was plated on marine broth (MB) agar for 3 days at 30°C. Out of 24 randomly selected colonies from the MB agar plates, 8 were identified as strain TOML190 via 16S rRNA gene analysis and were cryopreserved. A single colony was revived in liquid MB and cultivated with agitation at 30°C, and genomic DNA was extracted using a DNeasy UltraClean microbial kit (Qiagen). A genomic library was prepared using the rapid barcoding kit (Oxford Nanopore Technologies [ONT]), and sequencing was performed on a MinION Mk1B device with a FLO-MIN106D flow cell. The raw sequencing data were base called with Guppy v4.3.4 (ONT) using the high-accuracy mode, and the length and quality score of the sequences were obtained using NanoPlot v1.36.1 (6). The sequenced reads were filtered (length, >1,000 bp; Q-score, >12) using NanoFilt v2.5.0 (6). In total, 144,684 reads (average length, 3,405 bp) were obtained.

*De novo* assembly was performed using Flye v2.8.3 with –iterations 3 and –plasmids as options (7), and polishing was performed using Medaka v1.3.2 (ONT). The assembled genome was a single linear contig of 2,454,686 bp with GC content of 43.4% and 50× genome coverage. No plasmids were identified. BUSCO assessment (v5.2.2) was conducted against the *Oceanospirillales* lineage data set (8) to validate the quality of the genome. The BUSCO score (92.6%) and the genome length showing similarity to other *Kangiella* strains suggest that a near-complete draft genome sequence was attained. Gene annotation was performed using the RAST server (9), resulting in 2,332 coding sequences. Finally, annotated genes were analyzed using KofamKOALA (10), and metabolic pathways were identified using KEGG Mapper (11).

Features of strain TOML190 were highlighted by comparing its genome with those of other *Kangiella* species from the KEGG database. Primary pathway comparison showed that it lacks the sulfate permease and harbors a biofunctional sulfate adenylyltransferase-adenylylsulfate kinase. It also harbors genes (*cyoABCDE*) encoding a cytochrome *o*-ubiquinol oxidase that are expressed at high oxygen levels (12). These genes were not identified in currently reported *Kangiella* strains. The elucidated features of strain TOML190 suggest that it showed possible signs of adaptation to its residing host. We believe that *Kangiella* sp. TOML190 may serve as an important candidate to further understand the role of *Kangiella* in symbiotic settings.

### Data availability.

This whole-genome sequencing project and the 16S rRNA gene sequence of *Kangiella* sp. TOML190 have been deposited at DDBJ/EMBL/GenBank under the accession numbers BQYL01000001 and LC707240, respectively. The ONT base-called FASTQ files are available in the NCBI Sequence Read Archive (SRA) under the accession number DRA014096.
